# Breast cancer knowledge and practice of breast self‐examination among Palestinian female, West Bank: A cross‐sectional study

**DOI:** 10.1002/hsr2.1678

**Published:** 2023-11-01

**Authors:** Afnan W. M. Jobran, Mohamad A. Banat, Bashar Yaser Awad, Haya J. Warasna, Yasmeen R. Taqatqa, Mahmoud Jawabreh, Yasmeen R. Abualrub, Muhamad Zakaria Brimo Alsaman, Tarek A. Owais, Saif Salman

**Affiliations:** ^1^ Faculty of Medicine Al Quds University Jerusalem Palestine; ^2^ Faculty of Medicine Palestine Polytechnic University Hebron Palestine; ^3^ Vascular Surgery Department Al‐Razi Hospital Aleppo Syria; ^4^ Faculty of Pharmacy Benisuef University Benisuef Egypt; ^5^ Departments of Neurological Surgery, Neurology and Critical Care Mayo Clinic Jacksonville Florida USA

**Keywords:** awareness, breast cancer, female, Palestine, West Bank

## Abstract

**Background:**

Worldwide, breast cancer (BC) is the most frequently discovered tumor in women. Breast self‐examination (BSE) is a helpful screening method that gives women more control over their bodies by educating them about the structures of their breasts and assisting in the early detection of any developing breast abnormalities. The purpose of this research was to assess Palestinian girls' degree of BSE awareness and practice.

**Method:**

A self‐administered questionnaire was used to gauge participants' knowledge about BC and associated topics. Through an online survey, all girls above the age of 20 are encouraged to take part in the study. Female university students at academic levels I, II, III, and IV in Palestine were also invited to participate in the study by way of an online survey.

**Results:**

The study included 467 female participants, with 69% of the individuals being single. The majority of females (68.7%) scored poorly on knowledge of BC disease (possible risk, methods of detection, methods of diagnosis, methods of treatment, signs and symptoms, information about mammography, and other knowledge questions), whereas only 31.7% scored well.

**Conclusion:**

BC, which is thought to be the most common malignant development among them and the second leading cause of cancer mortality, is one of the issues that women in the West Bank face. Screening methods are crucial for the early detection of BC and for lowering disease‐related morbidity and mortality. It has been advised that starting at age 20, every woman should take the BSE.

## 1 INTRODUCTION

Among women worldwide, breast cancer (BC) is the most often diagnosed cancer.^1^ With an anticipated 2.3 million cases and 685,000 fatalities from BC in 2020, it will surpass lung cancer as the most often diagnosed cancer and move up to the fifth spot in the global cancer death toll.^2^ By 2070, there will be 4.4 million cases, according to projections.^3^ According to the Palestinian Ministry of Health, BC is the most common cancer among women in Palestine, accounting for 16% of all cancer cases and having a mortality rate of 9.8% among those who develop the disease. In Palestine, there were 526 instances of BC reported in 2020.^2^, ^4^ 38.4 cases per 100,000 females are found in the West Bank. BC accounts for 31.3% of all cases of cancer among Palestinian women recorded in Gaza; its frequency is 149.1 per 100,000.^5^

The underlying premise of BC screening is to discover cancer in early stage, before they become palpable. The screening test is done on asymptomatic patients with high risks, and will not benefit all women who are diagnosed with BC. It's important in reduction of morbidity, mortality, and in early detection of BC.^6^ In 1997, the American Cancer Society updated the BC screening guidelines and recommended that “women should begin mammography's screening at 40 year annually,” also cessation of annual screening is not age related.^7^

A tiny tumor at an early stage of BC's progression is connected with a better prognosis and treatment response.^6^ One of the most important steps in creating prevention strategies for BC is identifying its risk factors. Older age, a high body mass index or obesity, tobacco use, physical inactivity, a high‐fat diet, early menarche, a late age at the first full‐term pregnancy, shorter breastfeeding intervals, hormonal menopausal therapy or oral contraceptives use, breast density, and a family history of BC are the main risk factors for BC.^8^

Breast self‐examination (BSE) and breast health awareness are vital habits for all women worldwide. Understanding how their breasts evolve over time will help them understand what is normal for them and how to spot any abnormal changes.^9^ To reduce the burden of the disease and improve survival rates, early detection, diagnosis, and effective treatment of BC are essential.^10^ Because of this, knowledge of the risks and symptoms of BC plays a significant role in determining the incidence and course of the disease.^11^ For instance, if females are adequately informed about BC, they can contribute to lowering the prevalence of BC in their community and help avoid cancer in themselves.^12^ Therefore, the purpose of this study was to gauge Palestinian women's awareness of BC. Additionally, the connection between BSE usage and awareness of BC is investigated.

## 2 METHODS AND MATERIALS

### 2.1 Study design, setting, and period

A cross‐sectional design was used to assess the level of knowledge and practicing BSE among females who are 20 years old and more, in West Bank, Palestine. Data were collected from December 2021 to January 2022.

### 2.2 Participants

All females who are above 20 years old are invited to participate in the study by an online questionnaire. Female students studying in Palestine universities, academic levels I, II, III, and IV were also invited to participate in the study by online questionnaire.

### 2.3 Questionnaire

To save time and effort translating and constructing the questionnaire, we got in touch with a Palestinian researcher to get a questionnaire from a study that was comparable to ours. To facilitate both student access and our ability to classify and organize the data, we created an online questionnaire.

To make the goal of the study and the confidentiality of the information collected from the participating females clear, a consent form was included with the questionnaire.

The questionnaire was broken down into four sections. The first section, which has 13 questions, asks about sociodemographic factors like age, address, marital status, the number of people living in the home, occupation, economic status, attendance at prior training sessions, and family history of BC or other diseases related to the breast.

In the second section, 16 questions tested the knowledge of female university students on topics such the definition of BC, risk factors, symptoms, screening procedures, early detection and diagnosis techniques, BC stages, and treatment options. They were also questioned regarding their understanding and what they would do if they were to create BC.

The final section of the questionnaire asks 17 questions about women's awareness of BSE, CBE, and mammography. The definitions of terms, their significance, the ideal time to conduct these tests, and the frequency of doing these tests are among the topics covered in the questions.

Six questions are asked of the females in the fourth section on their understanding of BSE, the best approach to take BSE, how frequently they practice, how long it takes to administer the exam, and the primary barriers that impede women from taking the exam. Since practicing BSE is done once a month, this part is not reviewed during posttest 1.

The female respondents' knowledge of BC, screening procedures, and frequency of BSE usage were evaluated using the questionnaire.

### 2.4 Data analysis

All the statistical analysis was performed using R Statistical Software (v4.1.3; R Core Team 2022), we calculated the mean and standard deviation (SD) for all the variables.

The demographics data was represented by the mean and the SD of the continuous variable (age, no of children, and household members) and by the prevalence of the levels of the categorical variables (all the other variables).

The questions of knowledge and practice were coded as 1 for the correct answer and 0 for the incorrect one, the total score of each section was calculated and the levels were considered as (good knowledge\practice = >70%, low knowledge\practice <70%).

We performed two logistic regression models to determine the association between the demographic variables and the knowledge levels in the first model and practicing the BSE in the second one.

A Pearson's correlation matrix was computed for assessing the linear relationship between the knowledge level, practice level, and if the participant is practicing BSE.

A *p*‐value of ≤0.05 was considered significant.

## 3 RESULTS

### 3.1 467 females participated in the study

The mean and SD of age was (24.4 [8.4]). Roughly half of them live in Hebron city (48.7%), then a quarter of them live in Bethlehem (27.4%), and the rest of the females are living in the other cities inside the West Bank, and the majority of them are not working (84%).

The prevalence of the single participants is (69%).

The illiterate percentage among their fathers and mothers is (3.5 and 5.1), respectively.

Regarding the family history of BC, 30% of the females had a family history and only 23 females had previous breast problems (Table 1).

**Table 1:** Demographics data.

Label	Levels	All
Age	Mean (SD)	24.4 (8.4)
Residence	Alquds	20 (4.3)
	Bethlehem	126 (27.4)
	Gaza	2 (0.4)
	Haifa	1 (0.2)
	Hebron	224 (48.7)
	Jenin	44 (9.6)
	Nablus	10 (2.2)
	Ramallah	8 (1.7)
	Tulkarm	4 (0.9)
	Yatta	1 (0.2)
	Other	20 (4.3)
Marital status	Married	143 (30.8)
	Single	322 (69.2)
Children	Mean (SD)	2.1 (2.3)
Working status	Yes	70 (15.1)
	No	393 (84.9)
Mothers education level	Illiterate	24 (5.1)
	Primary school	41 (8.8)
	Preparatory school	58 (12.4)
	Secondary education	154 (33.0)
	University education	190 (40.7)
Fathers education level	Illiterate	16 (3.4)
	Primary school	53 (11.3)
	Preparatory school	64 (13.7)
	Secondary education	137 (29.3)
	University education	197 (42.2)
Income level	<5000	237 (59.5)
	5000−9999	133 (33.4)
	≥10,000	28 (7.0)
Household members	Mean (SD)	6.9 (2.4)
Having special room	Yes	313 (67.0)
	No	154 (33.0)
Previous brest problem	Yes	23 (4.9)
	No	443 (95.1)
Family history	Yes	162 (34.7)
	No	305 (65.3)
Previous education session	Yes	429 (91.9)
	No	38 (8.1)

Regarding knowledge about BC disease [potential risk, methods of detection, methods of diagnosis, methods of treatment, signs and symptoms, knowledge about mammography, and other knowledge questions], the majority of the females (68.7%) had a low knowledge score while (31.7%) had a good knowledge level.

The table shows that most of the females had a high knowledge level toward some topics like (methods of detection [70.9%], methods of treatment [77.3%], and the signs and the symptoms of BC [51.0%]) while most of the participants showed low knowledge level about the other BC topics (Table 2).

**Table 2:** The percentage of knowledge level toward different BC topics.

Label	Levels	All
Potential risk level	Good	94 (20.1)
	Low	373 (79.9)
Methods of detection level	Good	331 (70.9)
	Low	136 (29.1)
Methods of diagnosis level	Good	106 (22.7)
	Low	361 (77.3)
Methods of treatment level	Good	361 (77.3)
	Low	106 (22.7)
Signs and symptoms level	Good	238 (51.0)
	Low	229 (49.0)
Mammography level	Good	95 (20.3)
	Low	372 (79.7)
Other knowledge questions	Good	143 (30.6)
	Low	324 (69.4)
Total knowledge level	Good	146 (31.3)
	Low	321 (68.7)

Abbreviation: BC, breast cancer.

Regarding practice, only (4.1%) of the participants had a good knowledge about how to practice BSE and CBE, only (27.4%) know how to perform BSE, (42.6%) know how often the BSE should be done, while only (11.1%) know how often they shall do the CBE if they are 20−40 year old.

On the other hand, the participated revealed some barriers that hinder their practice of BSE. The most important barriers are not having a breast problem (34.4%), do not knowing how to do it (15.4%), and thinking they shall not do it (7.5%) (Table 3).

**Table 3:** The percentage of practicing BSE.

Label	Levels	All
Who performs breast self examination.	Self	395 (84.6)
	Health professionals	55 (11.8)
	Others	17 (3.6)
Breast self examination is important in detecting breast cancer early	SA	188 (40.3)
	A	200 (42.8)
	N	65 (13.9)
	D	9 (1.9)
	SD	5 (1.1)
At what age should a girl begin breast self examination	At the age 20 years	212 (45.4)
	21−30 years	89 (19.1)
	31−40 years	111 (23.8)
	More than 40 years	55 (11.8)
How often should breast self examination be done.	Once a month and on a regular basis	199 (42.6)
	Once 2 months and on a regular basis	19 (4.1)
	Once 3−5 months and on a regular basis	70 (15.0)
	Once annually and on a regular basis	46 (9.9)
	At any time and not on a regular basis	42 (9.0)
	I dont know	91 (19.5)
How often do you practice breast self examination	At any time and not on a regular basis	96 (45.5)
	Once a month and on a regular basis	60 (28.4)
	Once annually and on a regular basis	20 (9.5)
	Once 3−5 months and on a regular basis	23 (10.9)
	Once 2 months and on a regular basis	12 (5.7)
How do you perform breast self examination	Using one finger	17 (3.6)
	Using the pads of the three middle fingers	128 (27.4)
	Using the three middle fingers and the palm of the hand	168 (36.0)
	There is no certain procedure	154 (33.0)
What is the best time to do breast self examination	After starting the menstrual period	22 (4.7)
	After the end of the menstrual period	219 (47.0)
	At any time	49 (10.5)
	I dont know	176 (37.8)
If you do not practice breast self examination regularly then what are the reasons…more than one answer is possible…	I do not feel comfortable doing this	5 (1.4)
	I do not have a breast problem	123 (34.4)
	I do not have a special room	5 (1.4)
	I do not know how to do that	55 (15.4)
	I do not think i should	27 (7.5)
	I do not think it is necessary	7 (2.0)
	I'm afraid	20 (5.6)
	I'm busy	24 (6.7)
	Too frequent to practice	22 (6.1)
	Any other reasons	70 (19.6)
What is the clinical breast examination CBE	It is a medical breast examination performed by the woman herself	49 (10.5)
	It is a medical examination performed by a trained healthcare provider	322 (69.0)
	It is a self examination performed by the woman herself	42 (9.0)
	I dont know	54 (11.6)
How often CBE should be done in female aged 20−40 years	Once every 6 months	139 (29.8)
	Once every 1 year	148 (31.7)
	Once every 2−3 years	52 (11.1)
	I dont know	128 (27.4)
Do you know how often CBE should be done to a woman aged 40 years and more	Once every 6 months	252 (54.0)
	Once every 1 year	73 (15.6)
	Once every 2−3 years	11 (2.4)
	I dont know	131 (28.1)
Total practice level	Good practice	19 (4.1)
	Low practice	448 (95.9)

The first logistic regression model was performed to determine the association between the knowledge levels (high and low) and the demographic variables (age, marital status, children, working status, mothers' education level, fathers education level, income level, having a special room, previous breast disorder, and having previous education session).

The variables that showed significant association were (age, marital status, children, and father's educational level [university educated compared to illiterate]) while no other significant association could be detected out of the model (Table 4).

**Table 4:** The association between the knowledge levels and the demographic variables.

Dependent: knowledge level		0 (low)	1 (good)	OR (univariable)	OR (multivariable)
Age	Mean (SD)	24.4 (8.5)	24.5 (8.3)	1.00 (0.98−1.02, *p* = 0.906)	1.07 (1.02−1.14, *p* = 0.015)
Marital status	Married	107 (74.8)	36 (25.2)	‐	‐
	Single	212 (65.8)	110 (34.2)	1.54 (1.00−2.42, *p* = 0.055)	6.07 (2.33−16.77, *p* < 0.001)
Children	Mean (SD)	2.4 (2.3)	1.5 (2.1)	0.83 (0.71−0.95, *p* = 0.009)	0.85 (0.65−1.07, *p* = 0.170)
Working status	Yes	42 (60.0)	28 (40.0)	‐	‐
	No	275 (70.0)	118 (30.0)	0.64 (0.38−1.10, *p* = 0.100)	0.83 (0.35−2.00, *p* = 0.675)
Mothers education level	Illiterate	19 (79.2)	5 (20.8)	‐	‐
	Primary school	31 (75.6)	10 (24.4)	1.23 (0.37−4.44, *p* = 0.743)	0.66 (0.13−3.50, *p* = 0.621)
	Preparatory school	43 (74.1)	15 (25.9)	1.33 (0.44−4.55, *p* = 0.630)	1.61 (0.28−10.14, *p* = 0.599)
	Secondary education	113 (73.4)	41 (26.6)	1.38 (0.52−4.37, *p* = 0.548)	0.94 (0.18−5.15, *p* = 0.936)
	University education	115 (60.5)	75 (39.5)	2.48 (0.95−7.73, *p* = 0.083)	1.70 (0.34−9.67, *p* = 0.533)
Fathers education level	Illiterate	14 (87.5)	2 (12.5)	‐	‐
	Primary school	37 (69.8)	16 (30.2)	3.03 (0.73−20.71, *p* = 0.173)	2.39 (0.37−22.74, *p* = 0.392)
	Preparatory school	49 (76.6)	15 (23.4)	2.14 (0.52−14.63, *p* = 0.348)	1.68 (0.22−18.09, *p* = 0.633)
	Secondary education	103 (75.2)	34 (24.8)	2.31 (0.60−15.19, *p* = 0.284)	2.62 (0.40−25.13, *p* = 0.346)
	University education	118 (59.9)	79 (40.1)	4.69 (1.26−30.37, *p* = 0.045)	2.84 (0.44−27.57, *p* = 0.307)
Income level	<5000	170 (71.7)	67 (28.3)	‐	‐
	5000−9999	87 (65.4)	46 (34.6)	1.34 (0.85−2.11, *p* = 0.206)	1.42 (0.60−3.30, *p* = 0.420)
	≥10,000	17 (60.7)	11 (39.3)	1.64 (0.71−3.65, *p* = 0.230)	1.60 (0.30−8.71, *p* = 0.575)
Having special room	Yes	214 (68.4)	99 (31.6)	‐	‐
	No	107 (69.5)	47 (30.5)	0.95 (0.62−1.44, *p* = 0.808)	1.34 (0.49−3.53, *p* = 0.559)
Previous brest problem	Yes	17 (73.9)	6 (26.1)	‐	‐
	No	303 (68.4)	140 (31.6)	1.31 (0.53−3.69, *p* = 0.579)	1.21 (0.34−5.02, *p* = 0.777)
Previous education session	Yes	291 (67.8)	138 (32.2)	‐	‐
	No	30 (78.9)	8 (21.1)	0.56 (0.24−1.20, *p* = 0.161)	1.39 (0.38−4.87, *p* = 0.609)

The second logistic regression model was performed to determine the association between if the participant is practicing BSE and the same demographic variables.

The model showed a univariable significant association regarding age, marital status, working status, and having special room, while the other variables showed no significant association in light of our data (Table 5).

**Table 5:** Logistic regression analysis to determine the association between practicing breast self‐examination (BSE) and demographic variables.

Dependent: do you practice BSE regulary		0 (no)	1 (yes)	OR (univariable)	OR (multivariable)
Age	Mean (SD)	23.3 (7.5)	27.0 (9.8)	1.05 (1.03−1.07, *p* < 0.001)	1.03 (0.98−1.09, *p* = 0.234)
Marital status	Married	81 (56.6)	62 (43.4)	‐	‐
	Single	244 (75.8)	78 (24.2)	0.42 (0.27−0.63, *p* < 0.001)	0.54 (0.21−1.36, *p* = 0.193)
Children	Mean (SD)	1.9 (2.4)	2.3 (2.1)	1.06 (0.94−1.20, *p* = 0.337)	0.89 (0.70−1.12, *p* = 0.350)
Working status	Yes	32 (45.7)	38 (54.3)	‐	‐
	No	291 (74.0)	102 (26.0)	0.30 (0.17−0.50, *p* < 0.001)	0.36 (0.16−0.80, *p* = 0.013)
Mothers education level	Illiterate	14 (58.3)	10 (41.7)	‐	‐
	Primary school	28 (68.3)	13 (31.7)	0.65 (0.23−1.86, *p* = 0.419)	1.02 (0.26−4.00, *p* = 0.980)
	Preparatory school	34 (58.6)	24 (41.4)	0.99 (0.38−2.64, *p* = 0.981)	0.76 (0.16−3.71, *p* = 0.732)
	Secondary education	113 (73.4)	41 (26.6)	0.51 (0.21−1.26, *p* = 0.134)	0.57 (0.13−2.44, *p* = 0.447)
	University education	138 (72.6)	52 (27.4)	0.53 (0.22−1.29, *p* = 0.151)	1.03 (0.24−4.56, *p* = 0.964)
Fathers education level	Illiterate	9 (56.2)	7 (43.8)	‐	‐
	Primary school	40 (75.5)	13 (24.5)	0.42 (0.13−1.37, *p* = 0.144)	0.30 (0.06−1.57, *p* = 0.153)
	Preparatory school	50 (78.1)	14 (21.9)	0.36 (0.11−1.16, *p* = 0.082)	0.63 (0.12−3.47, *p* = 0.591)
	Secondary education	101 (73.7)	36 (26.3)	0.46 (0.16−1.37, *p* = 0.148)	1.08 (0.21−5.66, *p* = 0.923)
	University education	127 (64.5)	70 (35.5)	0.71 (0.25−2.06, *p* = 0.512)	0.68 (0.13−3.53, *p* = 0.638)
Income level	<5000	163 (68.8)	74 (31.2)	‐	‐
	5000−9999	96 (72.2)	37 (27.8)	0.85 (0.53−1.35, *p* = 0.493)	1.11 (0.48−2.51, *p* = 0.807)
	≥10,000	18 (64.3)	10 (35.7)	1.22 (0.52−2.73, *p* = 0.630)	1.30 (0.27−6.29, *p* = 0.743)
Having special room	Yes	206 (65.8)	107 (34.2)	‐	‐
	No	121 (78.6)	33 (21.4)	0.53 (0.33−0.82, *p* = 0.005)	1.12 (0.43−2.90, *p* = 0.822)
Previous brest problem	Yes	13 (56.5)	10 (43.5)	‐	‐
	No	313 (70.7)	130 (29.3)	0.54 (0.23−1.29, *p* = 0.155)	1.23 (0.36−4.40, *p* = 0.737)
Previous education session	Yes	297 (69.2)	132 (30.8)	‐	‐
	No	30 (78.9)	8 (21.1)	0.60 (0.25−1.28, *p* = 0.214)	0.42 (0.10−1.46, *p* = 0.202)

## 4 DISSCUSION

One of the challenges facing women in the West Bank is BC, which is considered to be the most prevalent malignant development among them and the second largest cause of cancer fatalities. Screening techniques are essential for the early identification of BC and reduce morbidity and mortality from disease. It has been suggested that every woman should take the BSE starting at age 20. According to our knowledge, this is the first study on BC awareness in the West Bank; prior studies evaluating women's knowledge and BSE practices in the West Bank have not been conducted.

Our study showed that 95.1% of participants had prior awareness about BC, that 48% had strong knowledge scores regarding BC risk factors, that 54% of female participants had good knowledge scores regarding BC signs and symptoms, and that 75.8% had good knowledge scores about mammography and CBE.

Compared to studies that conducted in Arab countries regarding BC knowledge/awareness about risk factor, signs and symptoms, mammography and CBE; another two cross‐sectional studies conducted in palatine, the first was national cross sectional included 5434 adult women from 11 governorates, it showed that 41.7% of participants had good awareness about BC symptoms. Lump or thickening in breast were the most frequent BC symptoms,^13^ the second study was in Gaza among 86 female students about BC knowledge, it showed 80.2% had good knowledge about BC and good scores about risk factors, signs and symptoms of BC. Regarding general knowledge about BC disease and methods of early detection and management, the knowledge scores was low <70%.^14^

Alsareii et al. conducted study about BC awareness among 300 female students in Saudi Arabia. The study showed good knowledge about BC and poor knowledge about signs of BC.^15^

A study in UAE by Rahman et al. among 241 undergraduate students showed that 50% of them had knowledge about BC risk factor and only 38% know about warnings signs and symptoms. He reported that there is level of BC awareness was better among medical student.^12^ Similar result was found in another study in UAE, that showed lack of knowledge, attitude, among females toward BC that lead to low practices of screening.^16^

Two studies in Yemen contained women who attend primary healthcare centers in 2016 and 2019, the results ranged from poor knowledge to satisfactory knowledge.^17^, ^18^ In addition, poor knowledge about BC among female imprisonment facility in Sudan.^19^

El Asmar et al. showed high level about BC symptoms among Lebanese females, low level of curability, and mammography is better practiced than either BSE or CBE. The main barriers to obtaining a mammogram were participants are afraid to find out something wrong, painful and high cost.^20^

Al‐Ismaili et al. showed poor knowledge level of BC symptoms, risk factors, screening methods among Omani female teachers.^21^

Students know about BC from various sources, the most well‐known sources being university studies, the social media, and health professional. This finding also was shown in previous studies in Gaza Egypt and Saudi Arabia.^14^, ^15^, ^22^

The results of our study showed that the students have a great knowledge about the signs risk factors and bad effect of BC on their lives. Moreover, the outcome isn't consistent with another study, which is done in Al‐Quds university ‐Palestine for non‐health related disciplines which showed there is poor knowledge about BC risk factor, signs risk, and prognosis of the disease.^23^ This study showed good knowledge about early detection techniques, mammography, U/S, and CBE, which is consistent with previous studies. However they have poor practice for mammogram to detect BC.^23^

Good level of BC knowledge is an important key to increase the survival rate because of early diagnosis. The worldwide BC awareness is still low especially in developing and poor countries, according to the largest‐scale systematic review that done in 2022 included 92 articles. This level of knowledge did not increase overtime, that requires exerting more efforts to help them knowing more about BC.^24^

According to Cochrane review, applying interventions contributed significantly to increase women awareness such as providing information about BC symptoms and signs or increasing women's confidence and recognizing changes.^25^ In addition, education programs played an important role in determinate breast symptoms and improve the knowledge.^13^

The majority of the females (84%) in our study have heard about BSE, and 42.6% knew the time to do BSE; however, only a third (30.6%) practice it regularly.

These results are similar to the female university students and women in Gaza, 96.5% of university students have heard about BSE, 69.8% knew the time to do BSE, and only 31.4% practice it regularly.^14^ BSE practice among women in Gaza is low, 40% of them have never practiced BSE and 76.7% of participants aware of BSE.^26^

Two studies in UAE; first one showed the 68.5% of female students in Sharjah heard about BSE, and few of them performed BSE regularly.^12^ The second study demonstrated that 46% of females in UAE performed BSE.^16^

In addition, few of females practice BSE regularly in Iraq, KSA, Yemen, and Sudan.^18^, ^19^, ^27^, ^28^

Despite the poor knowledge of BC symptoms among Omani teachers, about half 56.1% of them practiced BSE.^21^

The level of BSE practice was high among nursing students in Alexandria University due to curriculum development that contain illustrated guide to BSE.^22^

BSE is a concept that mentioned first time by a surgeon called Cushman Haagensen in 1950. His purpose was to diagnosis BC early so surgeons could treat it.^29^ Female who has done BSE showed significant reason urge them to do that mainly it was because it helps in early detection of BC and family history of BC. On the other hand, the majority of women who don't do BSE explained that the reason is due to absence of breast problems, fear from doing it, don't know the correct way to perform that, don't have the time, and don't think that they need to do BSE.^30^, ^31^

## AUTHOR CONTRIBUTIONS

**Afnan W. M. Jobran**: Conceptualization; investigation; methodology; project administration; resources; software; validation; visualization; writing—original draft; writing—review and editing. **Mohamad A. Banat**: Investigation; writing—review and editing. **Bashar Yaser Awad**: Investigation; writing—review and editing. **Haya J. Warasna**: Investigation; writing—review and editing. **Yasmeen R. Taqatqa**: Investigation; writing—review and editing. **Mahmoud Jawabreh**: Investigation; writing—review and editing. **Yasmeen R. Abualrub**: Investigation; writing—review and editing. **Muhamad Zakaria Brimo Alsaman**: Writing—original draft; writing—review and editing. **Tarek A. Owais**: Formal analysis; methodology; software; writing—review and editing. **Saif Salman**: Investigation; methodology; writing—original draft; writing—review and editing.

## CONFLICT OF INTEREST STATEMENT

The authors declare no conflict of interest.

## ETHICS STATEMENT

The study has been conducted in alignment with the known Ethical research and surveillance recommendations for emergencies and disasters. The study protocol was approved by the ethical committee at the Institutional Review Board of Palestine Polytechnic University, Ref no: KA/41/2022. Consent to participate was obtained from the participants via the survey as the research used an online platform. All participants were fully informed about the study's nature.

## TRANSPARENCY STATEMENT

The lead author Muhamad Zakaria Brimo Alsaman affirms that this manuscript is an honest, accurate, and transparent account of the study being reported; that no important aspects of the study have been omitted; and that any discrepancies from the study as planned (and, if relevant, registered) have been explained.

## Data Availability

All data generated or analyzed during this study are included in this article [Tables, Figures, and Supplementary data]. Original data set/raw data are available from the corresponding author on reasonable request.
